# Development of a Droplet Digital Polymerase Chain Reaction for Rapid and Simultaneous Identification of Common Foodborne Pathogens in Soft Cheese

**DOI:** 10.3389/fmicb.2016.01725

**Published:** 2016-10-28

**Authors:** Paola Cremonesi, Claudia Cortimiglia, Claudia Picozzi, Giulietta Minozzi, Michela Malvisi, Mario Luini, Bianca Castiglioni

**Affiliations:** ^1^Institute of Agricultural Biology and Biotechnology, National Research CouncilLodi, Italy; ^2^DeFENS, University of MilanMilan, Italy; ^3^PTP Science ParkLodi, Italy; ^4^DiMeVet, University of MilanMilan, Italy; ^5^Istituto Zooprofilattico Sperimentale della Lombardia e dell'Emilia RomagnaLodi, Italy

**Keywords:** cheese, ddPCR, detection, foodborne pathogens, qPCR

## Abstract

Dairy products can harbor various microorganisms (e.g., *Campylobacter* spp., *Salmonella* spp., *Listeria monocytogenes*, verocytotoxin-producing *Escherichia coli*) arising from animal reservoirs, and which can become important sources of foodborne illness. Therefore, early detection of food pathogens is crucial to prevent diseases. We wished to develop an accurate quantitative protocol based on a droplet digital polymerase chain reaction (ddPCR) involving eight individual TaqMan™ reactions to detect simultaneously, without selective enrichment, *Listeria* spp., *L. monocytogenes, Salmonella* spp., verocytotoxin-producing *E. coli* and *Campylobacter* spp. in cheese. ddPCR (a “third-generation PCR”) provides absolute quantification of target DNAs without requirement of a standard curve, which simplifies experimentation and data comparability. The accuracy, specificity and sensitivity of the developed ddPCR system were assessed using purified DNA from 50 reference pathogenic and non-pathogenic strains from international or Italian collections and analyzing soft cheese samples artificially contaminated with serial dilutions (from 4 × 10^6^ to 4 × 10^1^ CFU/g) of pure cultures from the American Type Culture Collection. Finally, the performance of our ddPCR system was compared by parallel testing with quantitative PCR: it gave higher sensitivity (10^2^ CFU/g for the *Listeria* spp. assay) without the necessity of a standard curve. In conclusion, this is the first ddPCR system developed for simultaneous detection of common foodborne pathogens in cheese using a single set of amplification conditions. As such, it could become a useful strategy for high-throughput screening of microorganisms to evaluate the quality and safety of food products.

## Introduction

Over the past three decades, the incidence of foodborne illnesses has increased dramatically to become a major public-health issue. The US Center of Disease Control and Prevention estimates that each year ≈48 million Americans (1 in 6) become ill, 128,000 are hospitalized, and 3000 die of foodborne diseases [Centers for Disease Control and Prevention (CDC), 2011]. In Europe in 2013, 5196 outbreaks of foodborne illnesses with 43,183 cases, 5946 hospitalizations, and 11 deaths were reported [European Food Safety Authority and European Centre for Disease Prevention and Control (EFSA and ECDC), 2015].

Foodborne illnesses are usually caused by consumption of food/drinking water contaminated with pathogenic bacteria, bacterial toxins, viruses, or parasites that invade the body *via* the gastrointestinal tract (where the first symptoms usually occur). Everyone is at risk, but the most severe consequences are for infants, the elderly, and people with a compromised immune system [European Food Safety Authority and European Centre for Disease Prevention and Control (EFSA and ECDC), 2015].

Among the bacteria that can contaminate food, some have an animal reservoir. Milk and dairy products can become contaminated during production and harbor various microorganisms (e.g., *Campylobacter* spp., *Salmonella* spp., *Listeria* spp., verocytotoxin-producing *Escherichia coli*, including *E. coli* O157) that can be important sources of foodborne diseases. Raw milk and raw-milk products are experiencing increasing market demand worldwide due to their alleged superior nutritional properties (Quigley et al., 2013). Therefore, it is necessary to: (i) establish the absence of pathogens or their toxins to ensure food safety; (ii) monitor the effectiveness of hygienic processing; and (iii) verify product quality and shelf-life stability. Hence, food safety is dependent upon rapid detection of these pathogens in foodstuffs through sensitive, fast and cost-effective technologies to prevent illnesses.

Beside conventional, laborious, and time-consuming culturing approaches, molecular methods with higher sensitivity and specificity have been developed. Such methods can be categorized into those based on nucleic acids (e.g., polymerase chain reaction (PCR), multiplex PCR, real-time PCR, nucleic acid sequence-based amplification, loop-mediated isothermal amplification, oligonucleotide DNA microarray), biosensors (electrochemical, optical, mass-sensitive) and immunologic (enzyme-linked immunosorbent assay, lateral flow immunoassay) (Mortari and Lorenzelli, 2014; Law et al., 2015).

*In vitro* amplification of nucleic acids *via* PCR remains the most widely applied method in research and clinical laboratories for the detection, identification, and enumeration of foodborne pathogens (Postollec et al., 2011). During the past decade, quantitative PCR (qPCR) has emerged as a method for rapid detection of foodborne pathogens in dairy microbiology due to its accuracy and precision (Fukushima et al., 2010). Several qPCR protocols have been applied to *Campylobacter jejuni* (Yang et al., 2003), *E. coli* O157 (Paul et al., 2013) and *Salmonella* spp. (Hein et al., 2006).

If the concentration of pathogens in complex biologic food matrices is very low, the quantification step of qPCR can affect the accuracy of template quantification considerably (Ramakers et al., 2003). To circumvent this problem, droplet digital PCR (ddPCR) has been considered. This approach partitions the sample into hundreds of millions of water-in-oil droplets before thermal cycling (McDermott et al., 2013). These droplets are monitored for positive amplification after endpoint PCR amplification using fluorescent target-specific hydrolysis probes (Floren et al., 2015). Until now, this method has been adopted for: routine analyses of genetically modified organisms in food and animal feed (Morisset et al., 2013; Gerdes et al., 2016); detection and quantification of pathogenic bacteria such as *Salmonella* spp., *Campylobacter jejuni* and *Listeria monocytogenes* in environmental water (Rothrock et al., 2013); exact quantification of different species in meat and processed meat products (Floren et al., 2015); monitoring the dynamics of microbial populations in soils with different population levels (Kim et al., 2014).

We wished to develop an accurate quantitative protocol based on ddPCR involving eight individual TaqMan™ reactions to detect simultaneously, without selective enrichment, *Listeria* spp., *L. monocytogenes, Salmonella* spp., verocytotoxin-producing *E. coli*, and *Campylobacter* spp. in cheese.

## Materials and methods

### Bacterial strains and growth conditions

Strains and culture conditions (culture media, temperature, incubation time) are listed in Table 1. Most of the bacteria tested originated from international (American Type Colture Collection; Deutsche Sammlung von Mikroorganismen und Zellkulturen; Collection of Institute Pasteur; Salmonella Genetic Stock Centre; Culture Collection, University of Göteborg, Sweden) and Italian collections.

**Table 1:** **List of target and non-target species with growth conditions**.

**Species**	**Strain^a^**	**Toxins type**	**Agar medium^b^**	**Enrichment broth^b^**	**Incubation time (h)**	**Temperature °C**
*Escherichia coli* O157:H7	ATCC 35150	*stx1, stx2, eae*	TSA	BPW	24 ± 2	37 ± 2
*Escherichia coli*	ATCC 11229		TSA	BPW	24 ± 2	37 ± 2
*Escherichia coli* O113:H21	ED22	*stx2*	TSA	BPW	24 ± 2	37 ± 2
*Escherichia coli* O26:H-	EF3	*eae*	TSA	BPW	24 ± 2	37 ± 2
*Escherichia albertii*	EscAlb (DeFENS)		TSA	BPW	24 ± 2	37 ± 2
*Escherichia blattae*	DSM 4481		TSA	BPW	24 ± 2	37 ± 2
*Escherichia fergusonii*	DSM 13698		TSA	BPW	24 ± 2	37 ± 2
*Shigella boydii*	DSM 7532		TSA	BPW	24 ± 2	37 ± 2
*Shigella flexneri*	DSM 4782		TSA	BPW	24 ± 2	37 ± 2
*Shigella sonnei*	ATCC 29930		TSA	BPW	24 ± 2	37 ± 2
*Shigella sonnei*	PO2		TSA	BPW	24 ± 2	37 ± 2
*Salmonella enteritidis*	SGSC 2378		HEA	BPW	24 ± 2	37 ± 2
*Salmonella enteritidis*	SGSC 2275		HEA	BPW	24 ±2	37 ± 2
*Salmonella enteritidis*	ATCC13076		HEA	BPW	24 ± 2	37 ± 2
*Salmonella typhimurium*	SGSC 1412		HEA	BPW	24 ± 2	37 ± 2
*Salmonella typhimurium*	ATCC13311		HEA	BPW	24 ± 2	37 ± 2
*Salmonella typhimurium*	ATCC 14028		HEA	BPW	24 ± 2	37 ± 2
*Campylobacter jejuni*	CCUG 6824		Skirrow	BB	48 ± 2	42 ± 2
*Campylobacter coli*	CCUG 11283iso		Skirrow	BB	48 ± 2	42 ± 2
*Campylobacter jejuni*	ATCC 33291		Skirrow	BB	48 ± 2	42 ± 2
*Campylobacter jejuni*	IZSLER		Skirrow	BB	48 ± 2	42 ± 2
*Campylobacter coli*	IZSLER		Skirrow	BB	48 ± 2	42 ± 2
*Campylobacter lari*	IZSLER		Skirrow	BB	48 ± 2	42 ± 2
*Campylobacter upsaliensis*	IZSLER		Skirrow	BB	48 ± 2	42 ± 2
*Campylobacter fetus*	IZSLER		Skirrow	BB	48 ± 2	42 ± 2
*Campylobacter hyointestinalis*	IZSLER		Skirrow	BB	48 ± 2	42 ± 2
*Listeria innocua*	263651/13		ALOA	TSB	24 ± 2	37 ± 2
*Listeria innocua*	DSM 20649		ALOA	TSB	24 ± 2	37 ± 2
*Listeria innocua*	ATCC 33090		ALOA	TSB	24 ± 2	37 ± 2
*Listeria innocua*	IZSLER		ALOA	TSB	24 ± 2	37 ± 2
*Listeria monocytogenes*	ATCC 13932		ALOA	TSB	24 ± 2	37 ± 2
*Listeria monocytogenes*	CIP 105449		ALOA	TSB	24 ± 2	37 ± 2
*Listeria monocytogenes*	IZSLER		ALOA	TSB	24 ± 2	37 ± 2
*Listeria ivanovii*	IZSLER		ALOA	TSB	24 ± 2	37 ± 2
*Staphylococcus aureus*	ATCC 23235		BP-RPF agar	BHI	24 ± 2	37 ± 2
*Bacillus cereus*	DSM 14579		CSA	BHI	24 ± 2	30 ± 2
*Streptococcus thermophilus*	BT 63		M17	M17	48 ± 2	37 ± 2
*Clostridium butyricum*	30		RCM	RCM	48 ± 2	37 ± 2
*Aeromonas hydrophila*	DSM30187		TSA	TSB	24 ± 2	37 ± 2
*Enterococcus faecalis*	ATCC 27332		m Enterococcus agar	m Enterococcus agar	24 ± 2	37 ± 2
*Enterococcus hirae*	ATCC 8043		m Enterococcus agar	m Enterococcus agar	24 ± 2	37 ± 2
*Hafnia alvei*	DSM 30163		TSA	TSB	24 ± 2	37 ± 2
*Klebsiella oxytoca*	KleOxy (DeFENS)		TSA	TSB	24 ± 2	37 ± 2
*Klebsiella oxytoca*	DSM 5175		TSA	TSB	24 ± 2	37 ± 2
*Morganella morganii sub. morganii*	DSM 30164		TSA	TSB	24 ± 2	37 ± 2
*Proteus mirabilis*	DSM 4479		TSA	TSB	24 ± 2	37 ± 2
*Serratia marcescens*	SerMar (DeFENS)		TSA	TSB	24 ± 2	37 ± 2
*Vibrio agarivorans*	DSM 13756		Marine Broth	Marine Broth	24 ± 2	37 ±2
*Vibrio parahaemoliticus*	DSM 10027		Marine Broth	Marine Broth	24 ± 2	37 ±2
*Streptococcus bovis*	V5458		M17	M17	24 ±2	37 ±2

a CIP, Collection of the Institute Pasteur (Paris, France); DSM, German Collection of Microorganisms and Cell Cultures (Braunschweig, Germany); ATCC, American Type Culture Collection (MD, USA); SGSC, Salmonella Genetic Stock Centre (Calgary, Canada); CCUG, Culture Collection, University of Göteborg (Göteborg, Sweden); DeFENS, Internal collection of Department of Food, Environmental and Nutritional Sciences, University of Milan; IZSLER, Internal collection of Istituto Zooprofilattico Sperimentale della Lombardia e dell'Emilia Romagna.

b ALOA, Agar Listeria Acc. To Ottaviani & Agosti; BAE, Blood Agar with Esculine; BB, Bolton Broth; BHI, Brain-Heart Infusion (Merck); BP-RPF agar, Baird Parker with Rabbit Plasma Factor; BPW, Buffered Peptone Water; CSA, Cereus Selective Agar; HEA, Hektoen Enteric Agar; M17 agar and broth; RCM, Reinforced Clostridium Agar; Skirrow, Skirrow selective medium; TSA, Tryptic Soy Agar; TSB, Tryptic Soy Broth (Merck); m Enterococcus agar (BD Difco™); Marine Broth (BD Difco™).

*E. coli* ED226 and EF3 strains were provided by Istituto Superiore di Sanità (Rome, Italy); *Shigella sonnei* PO2 is part of the Centro Enteropatogeni Italia Settentrionale (Milan, Italy) collection; *L. innocua* 263651/13 was isolated from an environmental sample from Istituto Zooprofilattico Sperimentale della Lombardia e dell'Emilia Romagna (Brescia, Italy), which also supplied *L. innocua, L. ivanovii, C. jejuni, C. coli, C. lari, C. upsaliensis, C. fetus*, and *C. hyointestinalis*. *Streptococcus thermophilus* BT63, *St. bovis* V5458 and *Clostridium butyricum* 30 were supplied by ISPA-CNR (Milan, Italy). *E. albertii* (isolated from lake water), *Klebsiella oxytoca* (isolated from fresh cheese) and *Serratia marcescens* (isolated from fresh cheese) were provided by the Department of Food, Environmental and Nutritional Sciences of the University of Milan.

All strains were cultivated aerobically except for *Campylobacter* spp., the isolates of which were grown under microaerophilic conditions. Stock cultures were thawed on selective agar plates; then single colonies were inoculated into appropriate enrichment broth for 24–48 h (Table 1). Five hundred microliters of each culture were used for DNA extraction.

### Spiking of food samples

*L. innocua* 263651/13, *S. typhimurium* ATCC 14028 and *E. coli* ATCC 35150 strains were used to contaminate soft cheese samples artificially to evaluate the performance of qPCR and ddPCR. Pure cultures of each bacteria type were grown for 24–48 h (as described above) and the concentration was determined by inoculation of the tenfold dilution series onto appropriate agar plates. Serial dilutions (10^8^–10^1^ CFU/mL for *L. innocua*; 10^9^–10^1^ CFU/mL for *S. typhimurium* and *E. coli*) in 0.9% NaCl (Sigma–Aldrich, St Louis, MO, USA) were prepared: 1 mL of each dilution was used to artificially contaminate 25 g of soft cheese. The latter was weighed in a 50-mL sterile Falcon tube (Orange Scientific, Belgium), then 1 mL of bacteria suspension added. The Falcon tube was vortexed for 10 s. Then, 5 g of contaminated samples was mixed with 45 mL of 2% (*w/v*) K_2_HPO_4_ buffer solution (Sigma–Aldrich) and homogenized in a Stomacher® paddle blender (PBI, Milan, Italy) for 60 s. A negative control (sample of uncontaminated cheese in sterile buffer) was included. After homogenization, 500 μL were subjected to DNA extraction.

### DNA extraction from pure cultures and from samples of spiked soft cheese

DNA was extracted from 500 μL of pure cultures and from the samples of spiked soft cheese according to our previous protocol (Cremonesi et al., 2006) starting from step 2. For artificially spiked samples, few modifications were applied to the protocol. Briefly, 300 μL of binding solution and 400 μL of lysis solution, washing solution, and ethanol solution were used. All centrifugations were carried out at 500 × g, with a final centrifugation of 550 × g. DNA was eluted in 100 μL of elution buffer. Quality and quantity of DNA were evaluated by spectrophotometric (NanoDrop Technologies, Wilmington, DE, USA) means at an absorbance of 260 and 280 nm, respectively. DNA was stored at −20°C.

### Probe design for PCR target genes

Candidate assay targets for the eight bacteria of interest were chosen on the basis of published data. The *yccT* gene (which codes for a conserved protein of unknown function) was chosen to identify *E. coli* and the closely related *Shigella* spp. (Clifford et al., 2012). For Shiga toxin-producing *E. coli* (STEC), two probes for shigatoxin1 (*stx1*) and shigatoxin 2 (*stx2*) were designed by considering the conserved region screened in the National Centre for Biotechnology Information. The *eae* (intimin) probe has been described by our research team (Cremonesi et al., 2014). The assay for *Campylobacter* spp. was designed on a specific region of the 16S rRNA gene to identify all the bacteria belonging to this species. For *Listeria* spp. and *Salmonella* spp., phosphoribosylpyrophosphate synthetase (*prs*) and invasion protein A (*invA*) were chosen because of their specificity for these species, respectively. The *L. monocytogenes* assay was designed on the *inlA* gene (which codes for a virulence protein that mediates adhesion and internalization into host cells).

After selection of target genes, specific target probes were designed using Primer Express® v3.0 (Applied Biosystems, Foster City, CA, USA) by setting the annealing temperature of primers and probes at 60 and 70°C, respectively. The nucleotide BLAST tool (https://blast.ncbi.nlm.nih.gov/Blast.cgi) was used to confirm the specificity of oligonucleotides *in silico*. Primers and TaqMan probes were synthesized by Applied Biosystems (Life Technologies Inc, Italy). Primers, 5′6-fluorescein-labeled (FAM) TaqMan probes, target genes, and reference sequences are listed in Table 2.

**Table 2:** **TaqMan™ assays used for qPCR and ddPCR**.

**Assay name**	**Target species**	**gene**	**Sequences (5′-3′)**	**Accession number**	**Amplicon (bp)**	
E.coli/Shig_yccT	*E. coli/Shigella* spp.	*yccT*	GCAGCGTGGTGGCAAAA^a^	CP010315	56	This study
			CGTGACCACCTTGATTGCAT^b^			
			CGGATACCGGCAAAC^c^			
STEC_stx1	*E. coli*	*stx1*	GGATTTCGTACAACACTGGATGATC	M16625	67	This study
			GATCAACATCTTCAGCAGTCATTACA			
			CAGTGGGCGTTCTT			
STEC_stx2B	*E. coli*	*stx2*	ACCCCACCGGGCAGTT	X07865	59	This study
			CGCGCCTGATAGACATCAAG			
			TTTTGCTGTGGATATACG			
STEC_eae	*E. coli*	*eae*	GTAACAATGTCAGAGGCGAGTTG	AE005174	73	Cremonesi et al., 2014
			CCACCGCTTGCTTTCAGTTTAA			
			ATTGCAGCCAAATATT			
Salmon_invA	*Salmonella* spp.	*invA*	TGGAAAGGGAAAGCCAGCTT	M90846	68	This study
			AATAGCGTCACCTTTGATAAACTTCA			
			ACGGTTCCTTTGACGGTG			
Camp_spp16S	*Campylobacter* spp.	16S	TTTTCGGAGCGTAAACTCCTTT	AB587657	66	This study
			GCCGGTGCTTATTCCTTAGGT			
			CTTAGGGAAGAATTCTG			
Liste spp._prs	*Listeria* spp.	*prs*	GGAGGCTGATTATGTCAAACGAGTA	CP002816	88	This study
			GCAATCTCTTCAGCTAGTTCACGAT			
			TTGATCCAAAGTTGAAGATT			
L.mono_inlA	*L. monocytogenes*	*inlA*	TAACAGACACGGTCTCGCAAA	CP013288	66	This study
			TCCCTAATCTATCCGCCTGAAG			
			AGATCTAGACCAAGTTACG			

a Primer forward.

b Primer reverse.

c TaqMan_Probe.

### qPCR

DNAs extracted from all pure cultures and from soft cheese contaminated artificially by several dilutions of *L. innocua, E. coli* and *S. typhimurium* were tested by qPCR. Reactions were carried out in 96-well plates sealed with adhesive optical covers (Applied Biosystems) and run on a QuantStudio™ 3 Real-Time PCR system (Applied Biosystems) at 2 min at 50°C, 10 min at 95°C, and 40 cycles of 15 s at 95°C and 1 min at 60°C. An identical thermal cycle was used for each target. All PCRs were done in duplicate. Each 20 μL of amplification reaction mix contained 1 μL of DNA (or water for negative controls), 10 μL of TaqMan Environmental Master Mix 2.0 (2 ×), 1 μL of TaqMan assay 20 × (18 μM for each primer, 5 μM for probe), TaqMan Exogenous Internal Positive Control (IPC) Reagents VIC™-labeled (2 μL of the ExoIPC Mix, Applied Biosystems), 0.4 μL of the Exo IPC DNA (target DNA) and 5.6 μL of molecular-grade water.

### ddPCR

DNA was detected and quantified using an QX100™ Droplet Digital™ PCR system (Bio-Rad Laboratories, Hercules, CA, USA). Reaction mixtures were set-up in a specific manner. Briefly, 10 μL of 2 × ddPCR Master Mix (Bio-Rad Laboratories) and 1 μL of TaqMan assay 20 × (18 μM for each primer, 5 μM for probe) were mixed with 1 μL of DNA from pure cultures, and nuclease- and protease-free water to complete a reaction volume of 20 μL. For samples of spiked food, a different amount of DNA template (2 μL for *E. coli* and 4 μL for *Salmonella* spp or *L. innocua* DNA) was used in the reaction mixture.

To generate the droplets, 20 μL of ddPCR and 70 μL of Droplet Generation oil for Probes (Bio-Rad Laboratories) were inserted in an eight-well cartridge using a QX100 droplet generator (Bio-Rad Laboratories) according to manufacturer instructions. Then, 40 μL of the generated droplet emulsion was transferred to a new 96-well PCR plate (Eppendorf, Hamburg, Germany) and amplified in a T100™ thermal cycler (Bio-Rad Laboratories). Amplification conditions started with 10 min of activation of DNA polymerase at 95°C, followed by 40 cycles of a two-step thermal profile of 15 s at 95°C for denaturation, and 1 min at 60°C for annealing and extension. A final hold of 10 min at 98°C was used for droplet stabilization followed by cooling to 4°C. No optimization of ddPCR was necessary with respect to qPCR annealing or probe concentration.

After thermal cycling, plates were transferred to a droplet reader (Bio-Rad Laboratories). The software provided with the ddPCR system (QuantaSoft 1.3.2.0; Bio-Rad Laboratories) was used for data acquisition to calculate the concentration of target DNA in copies/mL from the fraction of positive reactions using Poisson distribution analyses (McDermott et al., 2013) (Supplementary Table 2).

### Specificity and sensitivity

The specificity of each TaqMan assay was assessed using qPCR with purified genomic DNA from the reference strains described in Table 1. For each target assay, the DNA of other non-target bacteria was used as the negative control.

The limit of detection (LoD) for each qPCR and ddPCR assay was determined with pure culture, starting from 50 ng/μL of the DNA template, using a 100-fold dilution up to 5 fg/μL. The LoD for qPCR and ddPCR was also evaluated using soft cheese samples contaminated artificially by tenfold dilution from 4 × 10^6^ CFU/g up to 4 × 10^1^ CFU/g. Linearity over the dynamic range was determined by the coefficient of correlation (*R*^2^) calculated on the mean value of target copy numbers measured in the replicated dilution series for qPCR and ddPCR.

### Intra- and inter-assay repeatability

Repeatability was determined on a sub-sample of the TaqMan assay (STEC_eae, Salmon_invA, Liste spp_prs) using: (i) the DNA of three reference strains (50 pg/μL of *L. innocua* 263651/13, *S. typhimurium* ATCC 14028 and *E. coli* ATCC 35150); (ii) DNA samples extracted from artificially contaminated soft cheese (4 × 10^5^ CFU/g for each of the three types of bacteria); (iii) three DNAs extracted from artificially contaminated soft cheese (4 × 10^6^ CFU/g). Then, these sub-samples were mixed to form a pooled sample. For these tests, the same DNA was used as the technical replicate.

Intra-assay repeatability was assessed by calculation of the coefficient of variation (CV) of measured percentages from quadruplicate ddPCR measurements conducted in 1 day on a single sample run. The inter-assay test was evaluated by calculation of the CV of each sample, processed in duplicate for 5 consecutive days.

## Results

### Probe design

Each TaqMan assay, tested initially *in silico* through the BLAST tool, did not reveal identical sequences other than those targeted (100% of query cover and max identity). For verocytotoxin-producing *E. coli*, two assays (Table 2) were designed to detect virulence-specific genes such as *stx1* and *stx2*. The assay for detection of the intimin gene (*eae*) was taken from our previous data (Cremonesi et al., 2014).

### Assay specificity

The specificity of the eight TaqMan assays was assessed first by qPCR with 50 pathogenic target and non-target strains (Table 1). All trials identified the target strains correctly without generating false-positive or false-negative results, thereby confirming assay specificity. All TaqMan assays amplified their targets under identical qPCR conditions, and optimization was not done with ddPCR for annealing temperature or probe concentration. An identical protocol was used for qPCR and ddPCR, so the specificity test was not repeated for ddPCR.

### Assay sensitivity

#### Reference strains

For qPCR, the analytical sensitivity of all TaqMan assays tested in triplicate was ≈0.5 pg/μL of total DNA, with mean cycle threshold (C_*T*_) values from 28.9 ± 0.03 for *Campylobacter* spp. to 38.4 ± 0.91 for *Listeria* spp. (Table 3A). TaqMan assays for E. coli/Shig_yccT and *Campylobacter* spp. showed good sensitivity at 0.05 (33.8 ± 0.45) and 0.005 (35.2 ± 0.28) pg/μL, respectively.

Table 3**Sensitivity and efficiency of the TaqMan™ assays obtained by series of 100-fold dilutions of the pure culture genomic DNA (from 50 ng/μL up to 0.005 pg/μL; A) and with artificially contaminated soft cheese sample using tenfold dilution of 3 bacterial pure cultures (from 4 × 10^6^ CFU/g up to 4 × 10^4^ CFU/g; B) by qPCR and ddPCR**.

**Table:** 

**A: PURE CULTURE GENOMIC DNA**														
**qPCR**														
		**50 ng/μL**		**500 pg/μL**		**5 pg/μL**		**0.5 pg/μL**		**0.05 pg/μL**		**0.005 pg/μL**		
**Assay**	**Strain**	**Avg C_T_**	***sd***	**Avg C_T_**	***sd***	**Avg C_T_**	***sd***	**Avg C_T_**	***sd***	**Avg C_T_**	***sd***	**Avg C_T_**	***sd***	***R***^**2**^
*E.coli*/Shig_yccT	ATCC11229	16.1	0.06	22.4	0.03	28.6	0.01	31.8	0.12	33.8	0.45	Undetermined		0.98
STEC_stx1	ATCC35150	20.5	0.03	26.9	0.01	33.4	0.02	36.5	0.38	Undetermined		Undetermined		0.97
STEC_stx2B	ATCC35150	17.8	0.04	24.6	0.02	31.4	0.05	34.0	0.07	Undetermined		Undetermined		0.96
STEC_eae	ATCC35150	19.1	0.01	26.2	0.11	33.0	0.08	36.8	0.22	Undetermined		Undetermined		0.98
L.mono_inlA	CIP105449	^∧^		28.1	0.09	34.3	0.08	37.9	1.27	Undetermined		Undetermined		0.98
Liste spp._prs	263651/13	^∧^		26.9	0.07	33.9	0.04	38.4	0.91	Undetermined		Undetermined		0.98
Camp_spp16S	ATCC33291	13.0	0.01	19.2	0.01	25.7	0.04	28.9	0.03	32.4	0.34	35.2	0.28	0.98
Salmon_invA	ATCC14028	18.9	0.04	25.3	0.05	31.6	0.12	34.6	0.46	Undetermined		Undetermined		0.97
**ddPCR**														
		**50 ng/μL**		**500 pg/μL**		**5 pg/μL**		**0.5 pg/μL**		**0.05 pg/μL**		**0.005 pg/μL**		
**Assay**	**Strain**	**Avg copies/μL**	***sd***	**Avg copies/μL**	***sd***	**Avg copies/μL**	***sd***	**Avg copies/μL**	***sd***	**Avg copies/μL**	***sd***	**Avg copies/μL**	***sd***	***R*^2^**
E.coli/Shig_yccT	ATCC11229	^*^		1545	8.48	17.4	0.35	1.55	0.22	0.21	0.06	0.04	0.06	0.99
STEC_stx1	ATCC35150	^*^		1047	1.41	15	0.44	1.08	0.3	0.10	0.08	^§^		0.99
STEC_stx2B	ATCC35150	^*^		1270	30.4	19.7	0.72	1.15	0.23	0.09	0.08	^§^		0.99
STEC_eae	ATCC35150	^*^		1088	9.9	13.9	1.06	1.61	0.17	0.08	0.09	^§^		0.99
L.mono_inlA	CIP105449	^*^		1594	5.66	14.4	0.56	1.4	0.24	0.28	0.15	^§^		0.99
Liste spp._prs	263651/13	^*^		666	6.36	6.6	0.64	0.46	0.19	0.08	0.08	^§^		0.99
Camp_spp16S	ATCC33291	^*^		^*^	^*^	250	2.12	24.7	1.91	2.2	0.03	0.23	0.06	1
Salmon_invA	ATCC14028	^*^		1785	41.0	25.9	0.5	2.77	0.3	0.24	0.12	0.02	0.04	0.99

**Table:** 

**B: ARTIFICIALLY CONTAMINATED SOFT CHEESE SAMPLES**												
**qPCR**												
		**4 × 10^6^ CFU/g**		**4 × 10^5^ CFU/g**		**4 × 10^4^ CFU/g**		**4 × 10^3^ CFU/g**		**4 × 10^2^ CFU/g**		
**Assay**	**Strain**	**Avg C_T_**	***sd***	**Avg C_T_**	***sd***	**Avg C_T_**	***sd***	**Avg C***T*	***sd***	**Avg C_T_**	***sd***	***R***^**2**^
E.coli/Shig_yccT	ATCC11229	*nd*	*nd*	*nd*	*nd*	*nd*	*nd*	*nd*	*nd*	*nd*	*nd*	*nd*
STEC_stx1	ATCC35150	29.6	0.10	32.7	0.20	35.8	0.39	undetermined		undetermined		0.99
STEC_stx2B	ATCC35150	28.6	0.04	31.5	0.06	35.1	0.23	undetermined		undetermined		0.99
STEC_eae	ATCC35150	28.8	0.02	31.9	0.30	35.7	0.22	undetermined		undetermined		0.99
L.mono_inlA	CIP105449	*nd*	*nd*	*nd*	*nd*	*nd*	*nd*	*nd*	*nd*	*nd*	*nd*	*nd*
Liste spp._prs	263651/13	28.3	0.01	31.7	0.10	35.2	0.20	undetermined		undetermined		0.96
Camp_spp16S	ATCC33291	*nd*	*nd*	*nd*	*nd*	*nd*	*nd*	*nd*	*nd*	*nd*	*nd*	*nd*
Salmon_invA	ATCC14028	28.7	0.007	30.4	0.04	33.3	0.03	36.8	0.40	undetermined		0.98
**ddPCR**												
		**4 × 10^6^ CFU/g**		**4 × 10^5^ CFU/g**		**4 × 10^4^ CFU/g**		**4 × 10^3^ CFU/g**		**4 × 10^2^ CFU/g**		
**Assay**	**Strain**	**Avg copies/μL**	***sd***	**Avg copies/μL**	***sd***	**Avg copies/μL**	***sd***	**Avg copies/μL**	***sd***	**Avg copies/μL**	***sd***	***R***^**2**^
E.coli/Shig_yccT	ATCC11229	*nd*	*nd*	*nd*	*nd*	*nd*	*nd*	*nd*	*nd*	*nd*	*nd*	*nd*
STEC_stx1	ATCC35150	18.6	0.41	2.1	0.61	0.26	0.13	0.03	0.03	^§^		0.99
STEC_stx2B	ATCC35150	18.4	0.72	2.5	0.20	0.24	0.11	0.03	0.02	^§^		0.99
STEC_eae	ATCC35150	22.4	0.53	3.2	0.14	0.28	0.30	0.04	0.07	^§^		0.99
L.mono_inlA	CIP105449	*nd*	*nd*	*nd*	*nd*	*nd*	*nd*	*nd*	*nd*	*nd*	*nd*	*nd*
Liste spp._prs	263651/13	230	9.9	23.1	4.04	1.8	1.46	0.27	0.31	0.03	0.07	0.99
Camp_spp16S	ATCC33291	*nd*	*nd*	*nd*	*nd*	*nd*	*nd*	*nd*	*nd*	*nd*	*nd*	*nd*
Salmon_invA	ATCC14028	53.5	4.24	10.2	0.35	1.37	0.35	0.05	0.11	^§^		0.99

nd, not determined; Undetermined, signal comparable to background noise.

∧ = after the extraction, the DNA concentration was 5 ng/μL.

* = DNA concentration at which the signal of the assay was saturated (more than 20,000 copies in reaction mixture).

§ = value lower than the limit of instrument detection.

**Figure 1:**
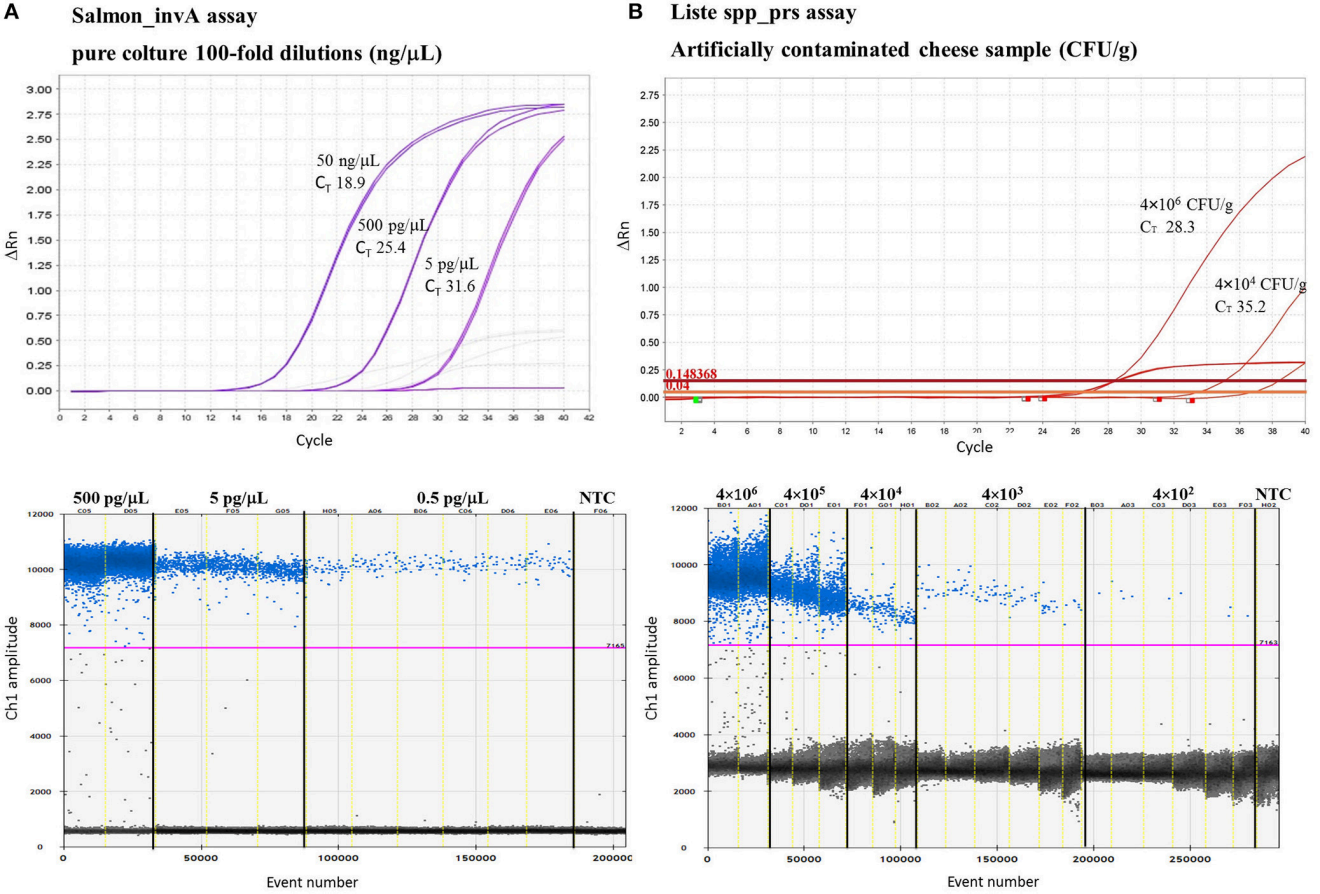
**Example of TaqMan™ assays analytical sensitivity by qPCR and ddPCR by using 100-fold dilution of DNA *S. enteritidis* SGSC2378 pure culture (A) and tenfold dilution of *L. innocua* 263651/13 in artificially contaminated soft cheese samples (B)**. For the qPCR (above) two replicates are shown while for ddPCR (1D Droplet Plots) up to six replicates were run with the lowest concentrations. NTC = negative template control.

To identify the lowest LoD in ddPCR, eight replicates were run with the two lowest concentrations of the DNA samples used to construct the standard curve. Good linearity was reached for all TaqMan assays revealing, with 0.05 pg/μL of total DNA, a mean of 0.08 ± 0.08 copies/μL for *E. coli eae* and Liste spp_prs assays and ≤ 2.2 ± 0.03 copies/μL for *Campylobacter* spp. Moreover, TaqMan assays for E. coli/Shig_yccT, *Campylobacter* spp. and *Salmonella* spp. showed good sensitivity for ≤5 fg of total DNA (0.04 ± 0.06; 0.23 ± 0.06; 0.02 ± 0.04 copies/μL, respectively) (Table 3B). TaqMan assays with qPCR and ddPCR showed good linearity in the range of quantification, with *R*^2^ of 0.96% and 1%, respectively. And more, with *Campylobacter* spp. assay, reaction saturation was reached at a concentration of 500 ng/μL (more than 20,000 positive droplets) and therefore it was impossible to quantify this concentration. The negative control for qPCR and ddPCR did not show amplification (data not shown). Examples of the results obtained are represented in Figure 1A and in Supplementary Figure 1.

#### Artificially contaminated soft cheese

To evaluate the performance of qPCR and ddPCR, soft cheese samples were contaminated artificially with a tenfold dilution series of three cultures of pure bacteria. Cheese samples spiked with verocytotoxin-producing *E. coli, L. innocua* and *S. typhimurium* showed good linearity within the range of quantification, giving *R*^2^ between 0.96 and 1 for qPCR and ddPCR, respectively. With qPCR and ddPCR, a sensitivity of 10^4^ CFU/g and 10^3^ CFU/g was reached for all the TaqMan assays tested. Moreover, the assays for *Salmonella* spp. and *Listeria* spp. showed good linearity at ≤10^3^ CFU/g and 10^2^ CFU/g, respectively. Examples of the results obtained are represented in Figure 1B.

### Inter- and intra-assay repeatability

For intra-assay experiments with the (i) DNA of three strains, (ii) three DNA samples extracted from artificially contaminated soft cheese (4 × 10^5^ CFU/g), (iii) DNA extracted from artificially contaminated soft cheese (4 × 10^6^ CFU/g) and then mixed in a pooled sample, the CV was 3.63, 10.41, and 10.62% for STEC_eae, 5.66, 7.73, and 3.44% for the Salmon_invA, and 10.8, 1.74, and 4.5% for Liste spp_prs, respectively. Inter-assay experiments showed a CV <12.99% and <15.91% for Salmon_invA and STEC_eae, respectively, and from 3.05 to 24.68% for Liste spp_prs (Supplementary Table 1).

## Discussion

Early detection of food pathogens is crucial to prevent foodborne illnesses. In the present study, eight individual TaqMan reactions were developed to detect *Listeria* spp., *L. monocytogenes, Salmonella* spp., verocytotoxin-producing *E. coli* and *Campylobacter* spp. directly and simultaneously in cheese. In a second step, a soft cheese was contaminated with three out of the five microorganisms under study.

After DNA extraction from cheese, an assay using a ddPCR instrument (a “third-generation PCR”) was developed to provide absolute quantification of target DNAs without the requirement of a standard curve. This procedure represents an important advantage in comparison with an assay based on qPCR because construction of a standard curve requires accurate quantification of the template DNA, which might be difficult to obtain (especially if working with food samples) (Kim et al., 2014). qPCR remains the most popular choice for the detection and quantification of a wide variety of microorganisms in food samples due to quantification of real samples, the shorter time required to obtain results, and lower costs (Hudecova, 2015). However, the presence of inhibiting substances decreases the efficiency of qPCR.

Given its advantages, the ddPCR system developed in the present study represents a new strategy to quantify pathogens directly in food samples, as described also by Floren et al. (2015) and Verhaegen et al. (2016). First, the ddPCR system optimized in the present study has increased the tolerance to inhibitors arising from cheese samples (e.g., fats, proteins, high concentration of Ca^2+^) to improve the LoD compared with qPCR. As reported by Rački et al. (2014) and Yang et al. (2014), this effect is probably due to partitioning of the PCR that reduces interference by PCR inhibitors (Huggett et al., 2013). Second, our approach was very effective when used for detection of DNA traces without the need for a pre-amplification step, and showed higher precision, sensitivity, and reproducibility over qPCR.

For the design of quantitative assays optimized in the present study, target genes described previously were used, such as the highly conserved region 16S rRNA for detection of *Campylobacter* spp., or bacterial virulence genes such as *stx1, stx2* and *eae* for verocytotoxin-producing *E. coli* (Verhaegen et al., 2016), *invA* for *Salmonella* spp., and *inlA* for *L. monocytogenes* (Rothrock et al., 2013). Using this strategy, good specificity and sensitivity were achieved.

For a quantitative protocol based on ddPCR developed in the present study, the dynamic range was comparable with qPCR. qPCR and ddPCR exhibited excellent linearity and efficiency, but ddPCR was more sensitive, improving the LoD in spiked cheese by one order of magnitude with respect to qPCR according to previous studies (Yang et al., 2014; Porcellato et al., 2016).

ddPCR was found to exhibit a saturation limit lower than that of qPCR, by which DNA samples must be diluted to a value <20,000 copies in the reaction mixture to quantify bacteria populated densely in a reference sample. As suggested by Yang et al. (2014), to determine the optimal dilution factor for ddPCR, the first step is the set-up TaqMan assays on qPCR using reference material. This statement was confirmed in our study by *Campylobacter* spp. assay that, because of its high efficiency, gave saturation signal at 500 pg/μl. When the artificially contaminated food samples were analyzed with ddPCR, no saturation was observed. This was probably due to the matrix effect on the efficiency of bacterial DNA extraction.

With this protocol sensitivity level, of 10^3^ CFU/g was reached for all the TaqMan assays (10^2^ CFU/g for *Listeria* spp.) in food matrices. These results could be improved or by a short selective enrichment of cheese sample or by the use of a higher efficiency DNA extraction method. Further studies should be necessary to evaluate new approaches.

Finally, although ddPCR is considered to be more expensive and time-consuming than qPCR (Verhaegen et al., 2016), its use to investigate simultaneously a sample for different pathogens, without standard curves, could reduce the difference in cost.

## Conclusions

Our results show the applicability of ddPCR to target the main foodborne pathogens in cheese. This technology is more sensitive for detection of low quantities of target DNA than qPCR, and reveals higher tolerance to inhibitors arising from food matrices. This is the first ddPCR system developed for simultaneous detection in cheese of common foodborne pathogens using a single set of amplification conditions. Hence, the good performance of this approach could be the starting point for becoming a useful approach for a high-throughput foodborne pathogens screening to evaluate quality and safety of the products. To be employed in routine testing, this ddPCR method shall be properly validated through intra-laboratories trials in order to demonstrate its efficiency.

## Author contributions

CP and ML provided, cultivated and characterized the strains and prepared the artificial contamination of cheese samples. PC and CC made the probes design and verify the “*in silico*” probe specificity; extracted the DNA from pure culture and artificially contaminated cheese samples. PC and CC performed part of the qPCR experiments and all the ddPCR experiments. GM and MM performed part of the qPCR experiments with the DNA extracted from pure culture strains. BC collaborated in ddPCR experiments and supervised the experimental study. PC, CC, CP, and BC drafted the manuscript. All the authors read, correct and approved the final manuscript.

### Conflict of interest statement

The authors declare that the research was conducted in the absence of any commercial or financial relationships that could be construed as a potential conflict of interest.

## Supplementary material

Supplementary Table 1 **Results obtained during the intra- and inter-assay repeatability**.

Supplementary Table 2 **dMIQE checklist for authors, reviewers and editors**.

Supplementary Figure 1 **Concentration plots show data for individual replicate wells across a dilution series**. Results of *Listeria* spp. assay, *L. monocytogenes* assay **(A)** *Campylobacter* spp. assay and *Salmonella* spp. assay **(B)** are reported. The error bars represent Poisson 95% confidence intervals.
