# Supplementary material for: Development of a Droplet Digital Polymerase Chain Reaction for Rapid and Simultaneous Identification of Common Foodborne Pathogens in Soft Cheese
Source: Front Microbiol. 2016 Oct 28;7:1725. doi: 10.3389/fmicb.2016.01725 (PMC5083709; doi:10.3389/fmicb.2016.01725)
Supplement: Supplementary Table 2 — dMIQE checklist for authors, reviewers and editors. [file Table2.DOC]

**Supplementary Table 2**: dMIQE checklist for authors, reviewers and editors.

| **Experimental design** | **Importance** | **Included** | **Comments** |
| --- | --- | --- | --- |
| Definition of experimental and control groups | E | Yes | see Materials and Methods |
| Number within each group | E | Yes | see Materials and Methods |
| Assay carried out by the core or investigator’s laboratory? | D | Yes | the core and investigator’s laboratory |
| Acknowledgment of authors’ contributions | D | Yes | see acknowledgment section |
| **Sample** |  |  |  |
| Description | E | Yes | see Materials and Methods |
| Volume/mass of sample processed | D | Yes | see Materials and Methods |
| Microdissection or macrodissection | E | No | n.a. |
| Processing procedure | E | No | n.a. |
| If frozen, how and how quickly? | E | No | n.a. |
| If fixed, with what and how quickly? | E | No | n.a. |
| Sample storage conditions and duration (especially for FFPEb samples) | E | Yes | All strains were stored at -20°C; cheese samples were analyzed immediately after spiking |
| **Nucleic acid extraction** |  |  |  |
| Nucleic acid quantification | E | Yes | only for DNA from reference strains |
| DNA or RNA quantification | E | Yes | DNA |
| Quality/Integrity, method/instrument, e.g. RNA integrity | E | Yes | only for DNA from reference strains |
| Template structural information | E | No | n.a. |
| Template modification (digestion, sonication, preamplification etc) | E | No | n.a. |
| Template treatment | E | No | n.a. |
| Inhibition dilutions or spike | E | No | n.a. |
| DNA contamination assessment of RNA samples | E | No | n.a. |
| Details of DNase treatment where performed | E | No | n.a. |
| Manufacturer of reagents used and catalogue number | D | Yes | see Materials and Methods |
| Storage conditions (Nucleic acid: temperature, concentration, duration, buffer) | E | Yes | stored at -20°C in Tris-EDTA (TE) buffer up to six months |
| **Reverse transcription (if necessary)** |  |  |  |
| cDNA priming method and concentration | E | No | n.a. |
| One or two-step protocol | E | No | n.a. |
| Amount of RNA used per reaction | E | No | n.a. |
| Detailed reaction components and conditions | E | No | n.a. |
| RT efficiency | D | No | n.a. |
| Estimated copies measured with and without addition of RT | D | No | n.a. |
| Manufacturer of reagents and catalogue numbers | D | No | n.a. |
| Reaction volume | D | No | n.a. |
| Storage conditions of cDNA | D | No | n.a. |
| **dPCR target information** |  |  |  |
| Sequence accession number | E | Yes | see Table 2 |
| Location of amplicon | D | No | n.a. |
| Amplicon length | E | Yes | see Table 2 |
| In silico specificity screen (BLAST, and so on) | E | Yes | see Materials and Methods and 3.1 paragraph in Results section |
| Pseudogenes, retropseudogenes, or other homologs? | D | No | n.a. |
| Sequence alignment | D | Yes | Available on request |
| Secondary structure analysis of amplicon | D | No | n.a. |
| Location of each primer by exon or intron (if applicable) | E | No | n.a. |
| What splice variants are targeted? | E | No | n.a. |
| **dPCR oligonucleotides** |  |  |  |
| Primer sequences | E | Yes | see Table 2 |
| RT Primer DB identification number | D | No | n.a. |
| Probe sequences | D | Yes | see Table 2 |
| Location and identity of any modifications | E | No | n.a. |
| Manufacturer of oligonucleotides | D | Yes | Life Technologies Inc, Italy for primers and probes |
| Purification method | D | Yes | HPLC |
| **dPCR protocol** |  |  |  |
| Complete reaction conditions | E | Yes | see Materials and Methods |
| Reaction volume and amount of cDNA/DNA | E | Yes | see Materials and Methods |
| Primer, (probe), Mg++ and dNTP concentrations | E | Yes | see Materials and Methods |
| Polymerase identity and concentration | E | Yes | ddPCR™ Supermix for Probes (No dUTP) from Bio-Rad |
| Buffer/kit identity and manufacturer | E | Yes | ddPCR™ Supermix for Probes (No dUTP) from Bio-Rad |
| Exact chemical composition of the buffer | D | No | Manufacturers proprietary information |
| Additives (SYBR Green I, DMSO, and so forth) | E | No | No additives |
| Manufacturer of plates/tubes and catalog number | D | Yes | Eppendorf twin.tec PCR Plate 96, semi-skirted blue plates (Thermo Fisher)/ 0030128605 |
| Complete thermocycling parameters | E | Yes | see Materials and Methods |
| Reaction setup (manual/robotic) | D | Yes | manual |
| Gravimetric or volumetric dilutions (manual/robotic) | D | Yes | manual |
| Total PCR volume prepared | D | Yes | see Materials and Methods |
| Partition number | E | Yes | Average n°droplet: 16700 |
| Individual partition volume | E | Yes | Corbisier et al., Anal Bioanal Chem (2015): 0.834nL |
| Total volume of the partitions measured (effective reaction size) | E | Yes | Number of partitions x 0.85 nL |
| Partition volume variance/SD | D | No | n.a. |
| Comprehensive details and appropriate use of controls | E | Yes | all NTC had 0% positive droplets |
| Manufacturer of dPCR instrument | E | Yes | Bio-Rad (Pleasanton, CA, USA) |
| **dPCR validation** |  |  |  |
| Optimisation data for the assay | D | No | the same TaqMan assays and temperature conditions of qPCR |
| Specificity (when measuring rare mutations, pathogen  sequences etc) | E | Yes | see Results |
| Limit of detection of calibration control | D | No | n.a. |
| If multiplexing, comparison with singleplex assays | E | No | n.a. |
| **Data analysis** |  |  |  |
| Mean copies per partition (λ or equivalent) | E | No | n.a. |
| dPCR analysis program (source, version) | E | Yes | see Materials and Methods |
| Outlier identification and disposition | E | No | No outliers identified |
| Results of NTCs | E | Yes | all NTC had 0% positive droplets |
| Examples of positive(s) and negative experimental results as supplemental data | E | Yes | see Figure 1 |
| Where appropriate, justification of number and choice of reference genes | E | No | n.a. |
| Where appropriate, description of normalization method | E | No | n.a. |
| Number and concordance of biological replicates | D | No | n.a. |
| Number and stage (RT or qPCR) of technical replicates | E | Yes | see Materials and Methods |
| Repeatability (intra-assay variation) | E | Yes | see Materials and Methods; see Results |
| Reproducibility (inter-assay/user/lab etc variation) | D | Yes | see Materials and Methods; see Results |
| Experimental variance or CI | E | Yes | see supplementary material |
| Statistical methods for analysis | E | Yes | see Materials and Methods |
| Data submission using RDML (Real-time PCR Data Markup Language) | D | No | n.a. |

All essential information (E) must be submitted with the manuscript. Desirable information (D) should be submitted if possible.

n.a.: not applicable
