# Supplementary figures and images for: Development of a Droplet Digital Polymerase Chain Reaction for Rapid and Simultaneous Identification of Common Foodborne Pathogens in Soft Cheese
Source: Front Microbiol. 2016 Oct 28;7:1725. doi: 10.3389/fmicb.2016.01725 (PMC5083709; doi:10.3389/fmicb.2016.01725)

# Liste spp.\_prs assay

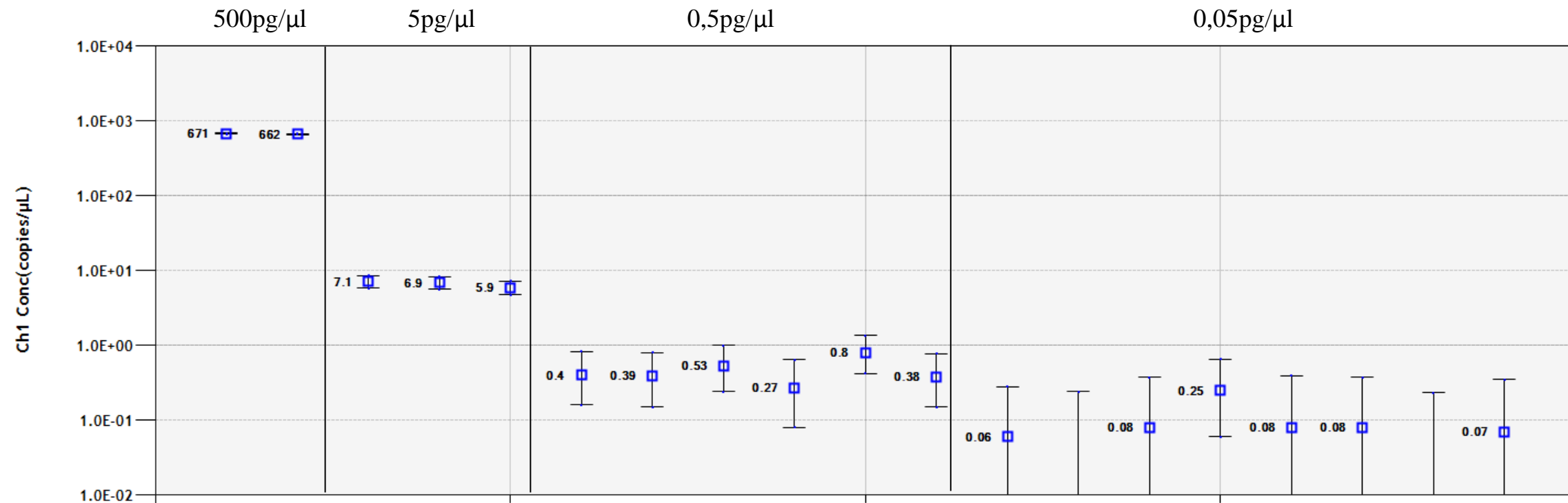

# L.mono\_inlA assay

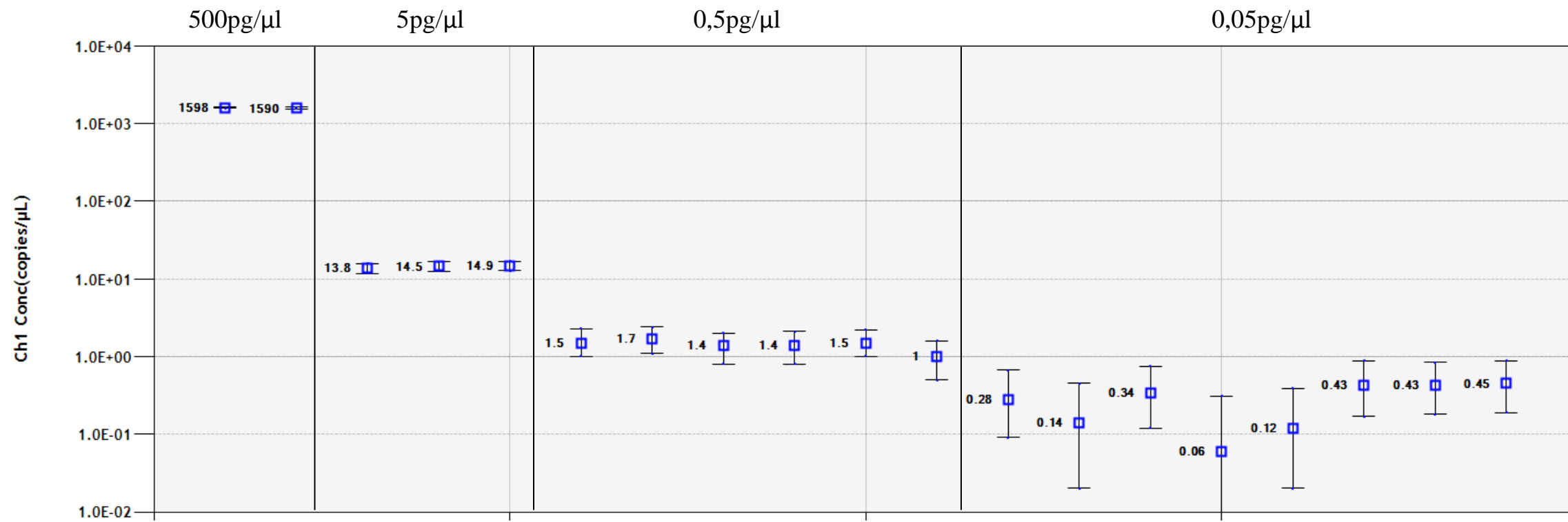

# Salmon\_invA assay

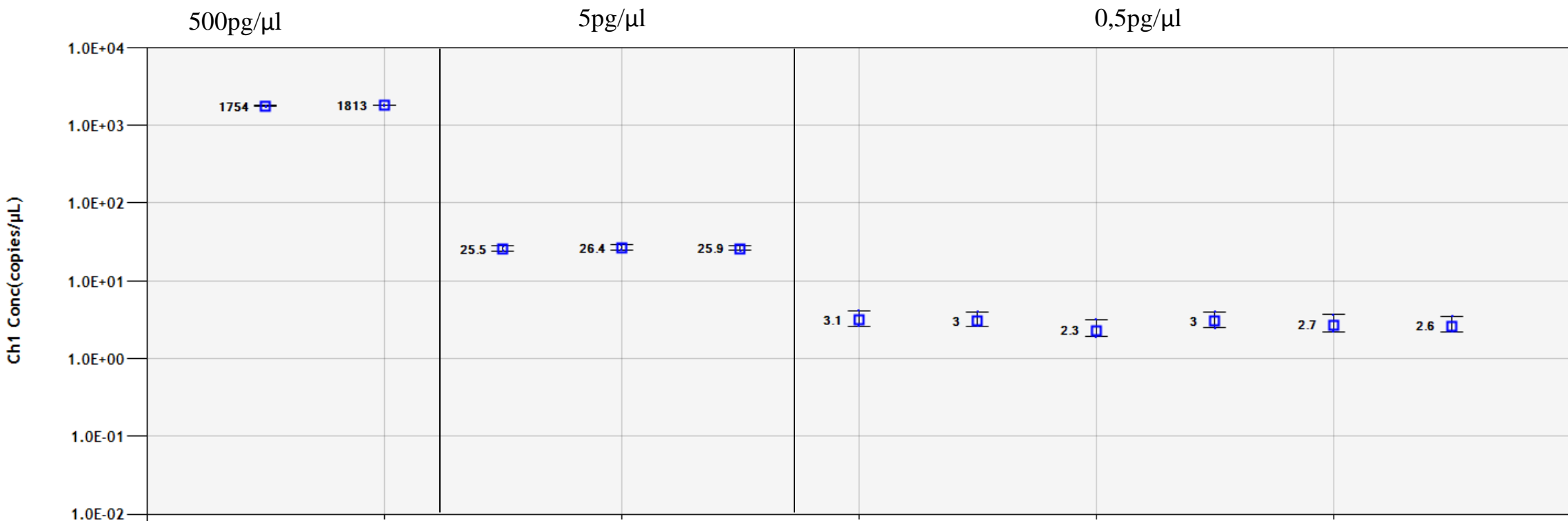

# Camp\_spp16S assay

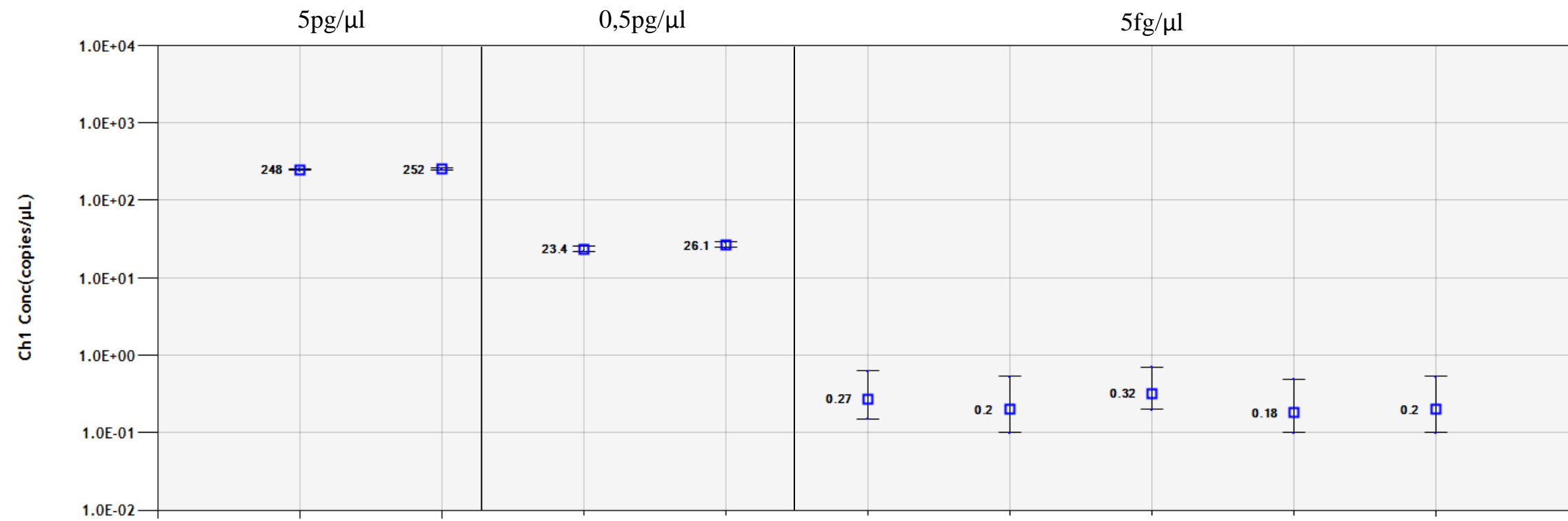

Supplement: Supplementary Figure 1 — Concentration plots show data for individual replicate wells across a dilution series. Results of Listeria spp. assay, L. monocytogenes assay (A) Campylobacter spp. assay and Salmonella spp. assay (B) are reported. The error bars represent Poisson 95% confidence intervals. [file Image1.PDF]
